# Bronchiolitis Obliterans With Recurrent Pneumothorax After Allogeneic Bone Marrow Transplantation

**DOI:** 10.7759/cureus.46633

**Published:** 2023-10-07

**Authors:** Masayuki Akatsuka, Naoya Yama

**Affiliations:** 1 Department of Intensive Care Medicine, Sapporo Medical University School of Medicine, Sapporo, JPN; 2 Department of Diagnostic Radiology, Sapporo Medical University School of Medicine, Sapporo, JPN

**Keywords:** anesthesia, perioperative, bone marrow transplantation, pneumothorax, bronchiolitis obliterans

## Abstract

Bronchiolitis obliterans syndrome (BOS) is a non-infectious pulmonary complication that can occur in patients who have undergone allogeneic bone marrow transplantation (BMT). BOS is characterized by the irreversible narrowing and obstruction of bronchi, resulting in severe respiratory distress and poor outcomes. This case report focuses on the complex management of a patient with a multifaceted medical history.

A 20-year-old man was initially diagnosed with precursor B lymphoblastic lymphoma and subsequently underwent allogeneic BMT. Nine months later, the patient was diagnosed with bronchiolitis obliterans with graft-versus-host disease, resulting in the development of BOS. Remarkably, 12 years after BMT, the patient was registered for lung transplantation. However, one year after registration, the patient developed a left pneumothorax. Despite rigorous efforts, including continuous thoracic drainage and autologous pleurodesis, the pneumothorax did not respond to treatment and required video-assisted thoracic surgery (VATS) bullectomy.

The preoperative assessment revealed a challenging clinical finding characterized by the need for home oxygen therapy (5 L/min with a nasal cannula), severe Hugh-Jones classification IV-V, and marked hypercapnia (partial pressure of carbon dioxide (pCO_2_), 76 mmHg). Imaging studies, including high-resolution computed tomography and chest radiography, revealed hyperinflation, emphysematous changes, and bronchiectasis across all lung lobes, further complicating the patient's condition.

Intraoperative management had the unique challenges of persistent hypoxia (P/F ratio 65-80), positive end-expiratory pressure of 5 cmH_2_O, and low tidal volumes (1.6-2.0 mL/kg) during one-lung ventilation. To address these problems, both-lung ventilation had to be performed intermittently. However, hyperventilation remained unmanageable, with maximum pCO2 values reaching 140 mmHg.

Following surgery, the patient had to be admitted to the intensive care unit in an intubated state. Fortunately, the following day, the patient's condition improved markedly, his state of consciousness was clear (Glasgow Coma Scale 15) and his pCO_2_ level improved (66 mmHg) with spontaneous breath. This course of events allowed extubation and the patient was discharged to the general ward only two days after surgery.

This case highlights the critical importance of a comprehensive preoperative assessment, including advanced imaging, when managing patients with BOS and complex pulmonary complications. Furthermore, it highlights the complexity and difficulty of perioperative respiratory management in such cases.

## Introduction

Bronchiolitis obliterans is a rare pulmonary complication that presents a formidable challenge in the field of hematopoietic stem cell transplantation (HSCT). Characterized by progressive and irreversible obliteration of the small airways [1], it imposes a substantial burden on post-transplantation patients. Although the pathogenesis of bronchiolitis obliterans is multifaceted, several contributing factors have been identified [2-4]. Allogeneic bone marrow transplantation (BMT), a cornerstone in the management of hematological malignancies, can introduce a complex interplay between host-versus-graft immune responses, graft-versus-host disease (GVHD), and conditioning regimens, all of which may potentiate the development of bronchiolitis obliterans [5].

While previous reports have delineated the clinical and pathological features of bronchiolitis obliterans [6,7], there persists a discernible lacuna concerning the concurrent manifestation of this disease and recurrent pneumothorax, as few cases have been documented in the literature [8,9]. The occurrence of pneumothorax in the context of bronchiolitis obliterans indicates an intricate interplay between the obliteration of small airways, increased airway resistance, and mechanical disruption of the lung parenchyma owing to alveolar damage [10].

In this case, we describe a patient who developed bronchiolitis obliterans after BMT. What makes this case unusual is that the patient experienced recurring episodes of pneumothorax, which is not a common complication typically associated with bronchiolitis obliterans following BMT. This atypical presentation challenges the conventional understanding of complications related to bronchiolitis obliterans. The interaction between these two conditions highlights the multifaceted nature of post-transplant pulmonary dysfunction, opening doors for further exploration and potential therapeutic interventions.

## Case presentation

The patient's medical history began with an initial diagnosis of precursor B lymphoblastic lymphoma, followed by allogeneic bone marrow transplantation (BMT). Nine months later, the patient received a diagnosis of bronchiolitis obliterans with graft-versus-host disease, leading to the development of BOS. Twelve years after the BMT, the patient was listed for a lung transplant. However, one year after being placed on the transplant list, the patient experienced a left-sided pneumothorax. Despite intensive efforts, which included continuous thoracic drainage and autologous pleurodesis, the pneumothorax did not respond to treatment and ultimately necessitated video-assisted thoracic surgery (VATS) for a bullectomy. The patient was a 20-year-old male (height 151.2 cm, weight 35.4 kg). The ASA physical status was 3. This patient received steroids and inhalation agents for BOS as pharmacotherapy.

Before the surgery, the preoperative assessment revealed a challenging clinical condition. The patient required home oxygen therapy at a rate of 5 liters per minute using a nasal cannula. The patient's respiratory status was notably severe, characterized by a Hugh-Jones classification of IV-V. Additionally, the patient displayed marked hypercapnia (pCO2 76 mmHg) along with a pO2 of 91 mmHg, a pH of 7.32, an HCO3- level of 38.6 mmol/L, and a BE of 12.2 mmol/L.

**Figure 1:**
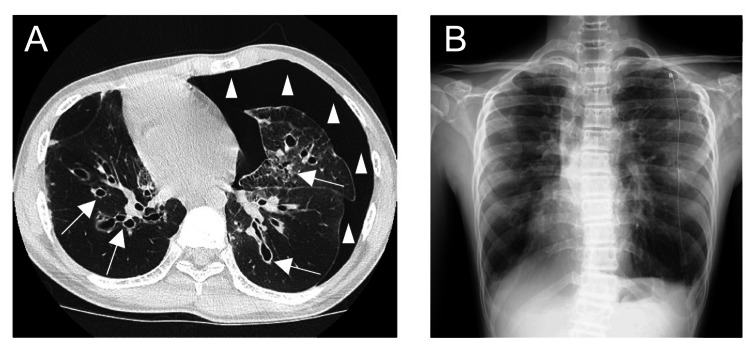
Computed tomography and chest radiograph These two images show that the right-sided deviation of the mediastinum is due to a left pneumothorax. (A) High-resolution computed tomography image showing hyperinflation, emphysematous changes, bronchiolitis obliterans (white arrows), and pneumothorax (white triangles). (B) Chest radiograph taken the day before surgery after insertion of a chest drain.

Advanced imaging studies, including high-resolution computed tomography and chest radiography, revealed hyperinflation, emphysematous changes, and bronchiectasis affecting all lung lobes (Figure 1). These findings added further complexity to the patient's condition.

The preoperative strategy involved planning for temporary bilateral lung ventilation to address hypoxemia during one-lung ventilation. This decision was made in consultation with the surgeon and included using low tidal volume ventilation with a maximum pressure set below 20 to minimize the risk of pneumothorax in the right lung.

We used inhalation anesthetic and intercostal nerve block during the surgery. Intraoperative management had the unique challenges of persistent hypoxia and low tidal volumes (1.6-2.0 mL/kg) during one-lung ventilation. To address these problems, both-lung ventilation had to be performed intermittently. However, hyperventilation remained unmanageable, with maximum pCO2 values reaching 140 mmHg. During the surgery, the patient had tachycardia of 100-120/min, systolic pressure of 100-110 mmHg, and mean arterial pressure of 65-70 mmHg.

Following surgery, the patient had to be admitted to the intensive care unit in an intubated state because the patient had hyperammonaemia and was difficult to extubate in the operating theater. Fortunately, the following day, the patient's condition improved markedly, his state of consciousness was good (Glasgow Coma Scale 15) and his pCO2 level improved (66 mmHg) with sufficient spontaneous respiration. This course of events allowed extubation and the patient was discharged to the general ward only two days after surgery.

This case underscores the vital significance of conducting a thorough preoperative assessment that incorporates advanced imaging in the care of patients dealing with BOS and intricate pulmonary issues. Moreover, it brings to light the intricate nature and challenges associated with perioperative respiratory management in such cases.

## Discussion

In this case report, we present a unique and challenging clinical scenario of a 20-year-old patient who developed BOS after allogeneic BMT for precursor B lymphoblastic lymphoma. The patient's condition was further complicated by recurrent pneumothorax, a rare co-occurrence that has not been extensively documented in the literature. Management of such cases presents significant perioperative challenges, particularly in terms of oxygenation and ventilation. Patients with BOS often have impaired gas exchange and reduced lung function. This makes maintaining adequate oxygen levels in the bloodstream a critical concern during surgery. Moreover, the compromised lung function in BOS patients can make ventilation management particularly complex. Achieving the right balance of ventilation, especially during one-lung ventilation, becomes a challenge. This includes setting appropriate tidal volumes, monitoring airway pressures, and avoiding barotrauma.

The complexity of BOS underscores the need for a thorough comprehension of the patient's respiratory condition before proceeding with surgical intervention. This understanding should encompass a detailed assessment of the patient's lung function, oxygenation, and ventilation capabilities to ensure a safe and tailored surgical approach. Previous studies have underscored the prognostic value of baseline pulmonary function tests (PFTs) for predicting postoperative outcomes in patients with bronchiolitis obliterans [11]. However, in the present case, this could not be performed due to the preoperative status of the patient.

CT and radiographic evaluations serve as invaluable tools for elucidating the structural alterations that characterize bronchiolitis obliterans [12]. These imaging modalities provide a comprehensive visualization of the pulmonary landscape, enabling clinicians to identify pathognomonic features, such as bronchiectasis and emphysematous changes, as were observed in our case. They also aid in gauging the extent of lung involvement and facilitate informed decision-making during surgery.

Although bronchiolitis obliterans and pneumothorax are known complications of HSCT and lung transplantation, respectively, their simultaneous occurrence in post-BMT patients remains a rare phenomenon. Previous studies have primarily focused on the individual aspects of these complications, such as the clinical and pathological features of bronchiolitis obliterans and the management of pneumothorax [13]. The coexistence of bronchiolitis obliterans and recurrent pneumothorax in our patient could be attributed to several factors. First, bronchiolitis obliterans, characterized by the progressive obliteration of small airways, leads to increased airway resistance and compromised lung function. This heightened airway resistance is likely to contribute to the development of pneumothorax, particularly when positive pressure ventilation is involved. In such cases, mechanical stress on the lung parenchyma can be exacerbated by alveolar damage associated with bronchiolitis obliterans. It's important to note that this relationship may not be as pronounced during spontaneous respiration. The pulmonary status of our patient was further complicated by the presence of GVHD.

This case report has inherent limitations. It represents a single patient's experience and, although it sheds light on a rare and complex clinical situation, it cannot provide generalizable conclusions. Furthermore, the specific management strategies employed in this case may not be universally applicable, thus, further investigation with larger cohorts is required. Additionally, long-term follow-up data are lacking and patient outcomes beyond the immediate postoperative period remain unknown.

## Conclusions

In summary, this case report offers a deeper understanding of the challenges and complexities associated with bronchiolitis obliterans syndrome and its concurrent occurrence with recurrent pneumothorax in post-BMP patients. It emphasizes the importance of comprehensive assessments, advanced imaging, and innovative management strategies in these complex cases, while also acknowledging the need for further research and long-term follow-up data.
